# HPV Testing for Cervical Cancer in Romania: High-Risk HPV Prevalence among Ethnic Subpopulations and Regions

**DOI:** 10.5334/aogh.2502

**Published:** 2019-06-20

**Authors:** Minodora Bianca Ilisiu, Dana Hashim, Trude Andreassen, Nathalie C. Støer, Florian Nicula, Elisabete Weiderpass

**Affiliations:** 1 Institute of Oncology “Prof. Dr. Ion Chiricuţa” of Cluj-Napoca: Prevention and cancer control Center, Cluj-Napoca, RO; 2 Department of Research, Cancer Registry of Norway, Institute of Population-Based Cancer Research, Oslo, NO; 3 Institute of Basic Medical Sciences, Faculty of Medicine, University of Oslo, Oslo, NO; 4 Oslo University Hospital, Norwegian National Advisory Unit on Women’s Health, Oslo, NO; 5 International Agency for Research on Cancer, Lyon, FR

## Abstract

**Background:**

Romania has had one of the highest rates of cervical cancer incidence and mortality in Europe for decades. Data on the high-risk human papillomavirus (hrHPV) prevalence within the Romanian population are crucial for cervical cancer intervention in high risk groups. The aim of this study was to determine the prevalence of hrHPV infection in Romania, identifying high-risk areas for cervical cancer prevention efforts.

**Methods:**

The target population of this study were women of all forms in Romania, including ethnic minorities, women from urban and rural areas, and women in various regions. Women with no history of precancerous or cancerous lesions were offered hrHPV screening. The specimens were tested with Hybrid Capture 2 (HC2) DNA test. Age-standardized hrHPV prevalence rates with 95% confidence intervals (CI) were estimated.

**Results:**

hrHPV results of 2060 women aged 18 to 70 years were analyzed. The highest hrHPV prevalence rates were observed among: Romanians (17.9%; 95 CI: 15.5–20.7%), Hungarians (16.6%; 95% CI: 13.1–20.8%), Russians (15.6%; 95% CI: 11.3–21.3%), women living in North (19.2%; 95% CI: 16.5–22.3%), and West regions (23.0%; 95 CI: 18.6–28.0%), and women living in urban areas (20.0%; 95 CI: 18.5–28.0%). hrHPV prevalence rates were lower for the Roma population (7.8%; 95% CI: 4.7–12.5%).

**Conclusions:**

These hrHPV prevalence rates in a high cervical cancer incidence country provide baseline information for targeted cervical cancer intervention strategies as well as a baseline to measure the impact of hrHPV vaccination in the future.

## Introduction

Cervical cancer is the fourth most common cancer type among women worldwide, and second most common in Romania [1]. Cervical cancer incidence and mortality rates in Romania are three times higher than in other European countries—19.9 and 8.9 per 100,000 women, respectively [1]. Current estimates indicate that every year, 3308 women in Romania are diagnosed with cervical cancer and 1743 die from the disease [2].

A population-based cervical cancer screening program was piloted in Romania from 2002 to 2008. The pilot program was conducted in Cluj County, and the program expanded to the entire northwest region four years later. In 2012, a nationwide population-based screening program was initiated by the Romanian Ministry of Health [3]. The program recommends free-of-charge cervical cytology every five years for women aged 25 to 64 years [2]. The coverage of the regional pilot program was 21% for all ethnical groups by the end of 2008 [4].

A major barrier in reducing cervical cancer screening in Romania has been the lack of a national cancer registry to monitor and record individual women’s screening history. Doctors and laboratories analyzing the screening tests are not obliged to report screening results to Romania’s seven regional cancer registries [5]. As high-risk Human Papillomavirus (hrHPV) is the main etiology of cervical cancers [2], data on high-risk HPV prevalence is the key to identifying high-priority areas to reduce the burden of cervical cancer in Romania [1,2]. However, hrHPV prevalence remains unknown for the population of Romania overall, as well as within large ethnic groups [2].

Over 10% of Romanian women is represented by non-ethnic Romanians [6]: 3.3% of those are estimated to be of Roma ethnicity although this number is likely underestimated [7]. Other ethnic groups, particularly the Roma population, participate less frequently in screening compared to the main population in Romania [8], which is linked to Roma women not knowing about the program’s existence, not believing the program is for free, and not believing that taking part in screening would lead to better health [9]. Further, there is no published data on the cervical health of other ethnic groups in Romania, including Roma, Hungarian-, Slovakian-, Ukrainian- and Russian-Romanian women. There is thus a knowledge gap regarding hrHPV prevalence among ethnic minorities in Romania.

Given the high incidence and mortality rates of cervical cancer in Romania and the lack of comprehensive data, the aim of this study was to determine the prevalence of hrHPV infection within Romania. Specifically, this study evaluates prevalence rates among Romanian sub-populations and regions, to identify high-risk areas for cervical cancer prevention efforts.

## Materials and Methods

### Participant selection

**Figure 1:**
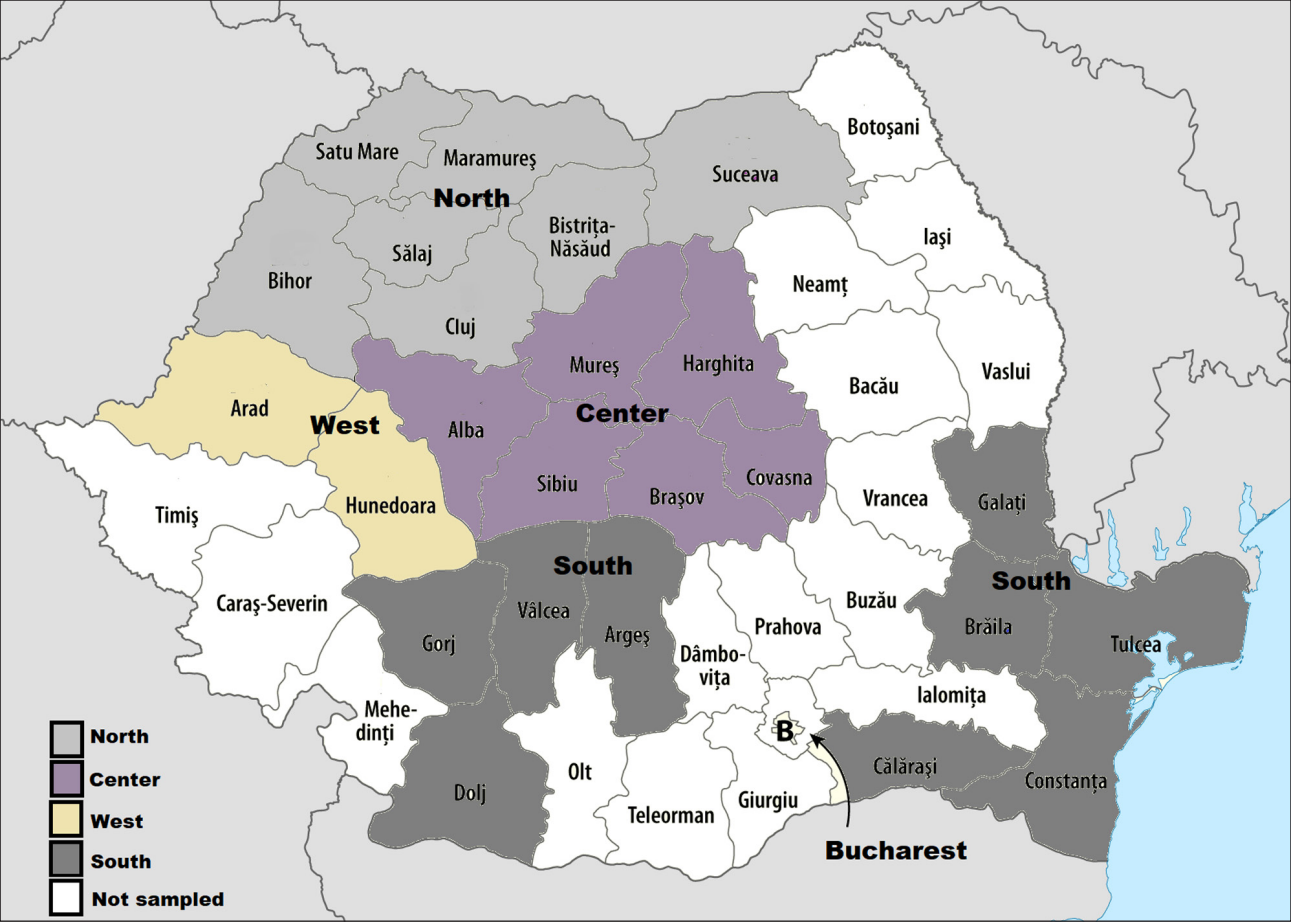
Regions of Romania from which patient population was sampled. Regions are defined by province: North (Satu Mare, Maramures, Salaj, Bistrita-Nasaud, Cluj, Bihor, Suceava), Center (Sibiu, Harghita, Mures, Alba, Brasov, Covasna), West (Munedoara, Arad), South (Oltenia [Dolj, Gorj, Valcea], Muntenia region [Calarasi, Arges], South-East [Tulcea, Braila, Constanta, Galati]).

The target population for hrHPV testing were women who were Romanian nationals, including women who self-identify as Romanian, Roma, Slovakian, Ukrainian, or Russians, and women from urban and rural remote areas (Figure 1). Women were contacted by their family doctors. To achieve high participation rate among ethnic minorities, information campaigns were conducted in rural and urban settlements with the aid of official staff members from the local mayor’s offices. Roma from isolated communities were contacted by Roma community leaders specially trained in health promotion and in communication with the general Romanian health sector.

Women agreeing to participate were questioned and examined by a single medical physician via face-to-face interview. From July 2015 to April 2017, data was collected on age, self-declared ethnicity, history of prior screening (yes or no), prior history of hysterectomy, and living area (urban [city] or rural). The inclusion criteria for participants were: women aged 18 to 70 years, self-identified Roma, Slovakian, Ukrainian or Romanian ethnicity, and urban or rural place of residence. Exclusion criteria were: history of cervical precancerous lesions or cancer, a complete hysterectomy, and history of adequate screening.

### Cervical screening program organization

Cervical screening test results from women in Romania are collected as part of the CEDICROM program (Improvement of Capacity of the Romanian Health Sector to Implement Organized Screening for Cancers). The CEDICROM project has been previously described [3,10] and was initiated at the Ion Chiricuta Institute of Oncology (IOCN), in the Department of Cancer Prevention and Control composed of the Northwest Regional Cancer and the Regional Management Unit cervical cancer screening program.

### hrHPV and cytology testing

The hrHPV tests were performed with Digene HC2 High-Risk HPV DNA Test (Qiagen, Gaithersburg, MD, USA, catalogue [cat.] number 5198–1220) detects the presence of 13 hrHPV types: 16,18, 31, 33, 35, 39, 45, 51, 52, 56, 58, 59, and 68. One technician in the IOCN was trained in HC2 methodology, which was standardized and validated by technicians from Digene. The collection method utilized a cervical Cyto PAP brush (SPL LIFE Science GMP Korea, cat. number 400200) to collect cells from the exo-/endo-cervix before dislodging the cells into a Thin Prep test vial liquid-based 20 ml PreservCyt Solution (Hologic, inc, USA, cat. number 234005) medium. Results of HC2 tests for HPV DNA were expressed as relative light units (RLU). Specifically, the ratio of the specimen light emission to the average emission of three concurrently tested positive controls (1 pg/ml HPV DNA) which corresponds to 5000 or more copies of HPV DNA [11]. Therefore, samples with a ratio value over 1 were considered hrHPV positive.

An endocervical brush was spread on the slide opposite the frosted end and sprayed with fixative, processed in one cytological laboratory (LEICA CV5030 autostainer XL). Cytology results were classified based on the 2001 Bethesda System [12] and agreed upon by two independent cytopathologists blinded to hrHPV test results. Cytological smears that were considered to be “positive” or abnormal tests were: atypical squamous cells (ASC-US) or worse low-grade squamous cell lesions (LSIL), atypical squamous cells cannot exclude HSIL (ASC-H), atypical glandular cells (AGC), and high-grade squamous intraepithelial lesion (HSIL) or cancer.

### Ethical approval

The study was approved in Romania by the Ethics Committee of the Institute of Oncology “Prof. Dr. Ion Chiricuţă” (IOCN) as part of its overall assessment of the project entitled “Cervical Cancer control among Roma and other disadvantaged groups of women” (CerCcRom). Assessment Record no. 28/10.12.2014, request no. 10988/10.12.2014. The following criteria of medical ethics were fulfilled: the informed consent of the study participants; protection and confidentiality issues concerning the personal data of the participants; balanced information on benefit and risk.

### Statistical analysis

Descriptive data of the study population are presented in frequencies. In order to exclude the differences in age structure between subpopulations, we standardized prevalence for age using the World Health Organization (WHO) standard population (2000–2025) for comparability [13] and calculated 95% confidence intervals (CI) based on the standard error (SE) of the proportion. Continuous variables were evaluated for normality and are presented as mean ± standard deviation (SD), using the ANOVA test to detect differences in means among groups. Contingency tables with the chi-square test or Fisher’s exact test, where appropriate, were used to compare differences in the proportions of hrHPV positive and abnormal cytology proportion by ethnicity, age group, region and urban (city) versus rural living area.

To evaluate the relationship between hrHPV positivity and ethnicity, region, urban vs. rural environment, and cytology result, we used logistic regression models to estimate odds ratios (ORs) and 95% CIs. To account for possible bias for women who had undergone cytology testing prior to the start of data collection, logistic regression analyses were also adjusted for prior cervical screening. All analyses were carried out using STATA version 14 (College Station, TX: StataCorp LLC).

## Results

### Overall

Between July 2015 to April 2017, 2060 women aged 18 to 68 years in Romania were hrHPV tested. Because almost half of the participants had never attended in screening previously (n = 955), these women underwent concurrent testing for hrHPV and abnormal cervical cytology within one visit according to Romanian national guidelines. Half of participating women were consecutively tested by a single physician in a mobile unit (MU) (1068; 51.8%), touring both rural and urban ethnically diverse regions of Romania: North (Satu Mare, Maramures, Salaj, Bistrita-Nasaud, Cluj, Bihor, Suceava), Center (Sibiu, Harghita, Mures, Alba, Brasov, Covasna), West (Munedoara and Arad), and South (Oltenia [Dolj, Gorj, Valcea], Muntenia region [Calarasi, Arges], South-east [Tulcea, Braila, Constanta, Galati]) (Figure 1). The other half of participating women were tested at regional hospitals (726; 35.3% public hospitals, 51; 2.5% private hospitals) or by family doctors (2015; 10.4%).

The demographic characteristics of 2060 total women are displayed in Table 1. The mean age of the participating women was 42.8 years (±9.7 SD). The overall age-standardized prevalence of hrHPV was 16.9% (95% CI: 14.7–19.5%). For age-specific prevalence, hrHPV infection was found to be the highest in the age groups of women younger than 34 years old (99; 23.1% hrHPV positive; chi-square p < 0.001). No significant difference was observed by age groups for abnormal cytology tests (chi-square p = 0.67).

**Table 1:** Demographic characteristics of the cervical cancer screened population in Romania, 2015–2017, n = 2060.

Age groups (years)	n	%

≤34	429	20,8
35–44	765	37,3
45–54	564	27,5
55+	297	14,4
Missing	5	0,2
Ethnicity		
Russian	149	7,2
Hungarian	63	3,1
Roma	124	6,0
Romanian	1615	78,4
Slovakian	85	4,1
Ukrainian	24	1,2
Region		
North (Satu Mare, Maramures, Salaj, Bistrita-Nasaud, Cluj, Bihor, Suceava)	892	43,3
Center (Sibiu, Harghita, Mures, Alba, Brasov, Covasna)	473	23,0
West (Munedoara, Arad)	186	9,0
South [Oltenia (Dolj, Gorj, Valcea), Muntenia region (Calarasi, Arges), South-East (Tulcea, Braila, Constanta, Galati)]	509	24,7
hrHPV Examination location		
Mobile unit	1068	51,8
Public hospital	726	35,2
Private hospital	51	2,5
Family physician	215	10,4
Rural or urban living area		
Rural	919	44,6
Urban (cities)	1141	55,4
Cytology test results*		
NILM	833	40,4
ASC-US	58	2,8
ASC-H	31	1,5
AGC-NOS	21	1,0
LSIL	4	0,2
HSIL	5	0,2
Unsatisfactory	3	0,2
Previously taken prior to study	1105	53,6

Abbreviations: ASC-US – Atypical squamous cells of undetermined significance; ASC-H – Atypical squamous cells – cannot exclude HSIL; LSIL – Low grade squamous intraepithelial lesion; HSIL – High grade squamous intraepithelial lesion; AGC – Atypical Glandular Cells not otherwise specified; NILM – negative for intraepithelial lesion or malignancy; hrHPV – high-risk Human Papillomavirus.

### Ethnicity

Women described themselves as Romanians (1615; 78.4%), Russians (149; 7.2%), Roma (124; 6.0%), Slovakians (85; 4.1%), Hungarians (63; 3.1%), and Ukrainians (24; 1.2%). Hungarian and Roma women had a younger mean age compared to other ethnic groups (aged 40 years for both groups, chi-square p = 0.01) (Supplementary Table 1).

Examining ethnicities and prior screening locations showed that the majority of Romanian women (701; 43.4%) were screened at public hospitals, while the majority of Russian (701; 43.4%), Hungarian (37; 58.73%), and all Ukranian women (24; 100%) were previously tested by MU (53.8%), while Roma (88; 70.9%) and Slovakian women (85; 100%) were screened by their family doctors (Supplementary Figure 1).

After standardizing for age, we found that the hrHPV prevalence was highest among Romanian women (17.9%; 95% CI: 15.5–20.7%), followed by Hungarian women (16.6%; 95% CI: 13.1–20.8%) and Russians (15.6%; 95% CI: 11.3–21.3%) (Table 2). The hrHPV prevalence was lowest among Ukrainian women (2.8%; 95% CI: 0.6–9.7%) compared to the other ethnic groups.

**Table 2:** HPV-positive prevalence rates by demographic characteristics in cervical cancer screening population in Romania.

	Crude			Age-adjusted*		

	Prevalence (%)	95% CI		Prevalence (%)	95% CI	

Ethnicity						
Romanian	15,5	13,8	17,3	17,9	15,5	20,7
Russian	16,8	11,2	23,8	15,6	11,3	21,3
Slovakian	12,9	6,6	22,0	13,1	9,0	18,7
Hungarian	14,3	6,7	25,4	16,6	13,1	20,8
Roma	6,5	2,8	12,3	7,8	4,7	12,5
Ukrainian	4,2	0,1	21,1	2,5	0,6	9,7
Regions						
North	18,0	15,6	20,7	19,2	16,5	22,3
Center	12,3	9,4	15,6	13,0	9,9	17,0
West	14,0	9,3	19,8	23,0	18,6	28,0
South	11,8	9,1	14,9	13,2	10,0	17,3
Environment						
Rural	14,2	12,0	16,6	14,8	12,4	17,7
Urban	15,2	13,2	17,5	20,0	16,8	23,6
HPV examination location						
Mobile unit	12,9	11,0	15,1	15,1	12,7	18,0
Public hospital	17,8	15,1	20,7	18,6	15,6	21,9
Private hospital	19,6	9,8	33,1	27,4	22,9	32,4
Family physician	13,0	8,8	18,3	17,4	12,6	23,5

* Adjusted for age according to WHO world standard population (2000 to 2025). Regions are defined by province: North: (Satu Mare, Maramures, Salaj, Bistrita-Nasaud, Cluj, Bihor, Suceava), Center (Sibiu, Harghita, Mures, Alba, Brasov, Covasna), West (Munedoara, Arad), South [Oltenia (Dolj, Gorj, Valcea), Muntenia region (Calarasi, Arges), South-East (Tulcea, Braila, Constanta, Galati)]. CI, confidence interval.

In logistic regression models adjusted for age, only Hungarian women had a lower likelihood of hrHPV positivity compared to Romanian women (OR = 0.37; 95% CI: 0.18–0.77) (Table 3). Because prior cervical cancer screening attendance was found to have an association with hrHPV positivity (OR = 1.93; 95% CI: 1.50–2.48), the likelihood of hrHPV positive status was also adjusted for. The association remained significant even after adjusting for prior cervical cancer screening attendance (OR = 0.44; 95% CI: 0.21–0.91).

**Table 3:** Correlates of hrHPV positivity for cervical cancer screened population in Romania.

	hrHPV positive	hrHPV negative	Age-adjusted			Adjusted for age and prior cervical cytology test		

	n	n	OR	95% CI		OR	95% CI	

Ethnicity								
Romanian	251	1364	1					
Russian	9	54	0,81	0,39	1,68	0,72	0,35	1,48
Hungarian	8	116	0,37	0,18	0,77	0,44	0,21	0,91
Roma	25	124	1,10	0,70	1,74	1,06	0,67	1,68
Slovakian	11	74	0,83	0,43	1,60	1,15	0,59	2,24
Ukrainian	1	23	0,22	0,03	1,65	0,30	0,04	2,24
Regionsa								
North	161	731	1			1		
Center	58	415	0,65	0,47	0,89	0,81	0,57	1,13
West	26	160	0,76	0,49	1,19	1,18	0,72	1,95
South	60	449	0,62	0,45	0,86	0,85	0,60	1,21
Rural vs urban living area								
Rural	131	788	1			1		
Urban	174	967	1,11	0,86	1,42	0,96	0,74	1,24
hrHPV examination locations								
Mobile unit	138	930	1					
Public Hospital	129	597	1,36	1,04	1,77	1,38	1,05	1,80
Private Hospital	10	41	1,62	0,79	3,32	1,66	0,81	3,44
Family Doctors	28	187	1,00	0,64	1,55	1,47	0,92	2,34
Cervical cytology resultb								
NILM	66	767	1			–	–	–
Low-grade	14	49	3,17	1,65	6,08			
High-grade	16	40	5,18	2,74	9,79			
Prior cervical cytology screening	209	897	2,54	1,89	3,41			

^a^ North (Satu Mare, Maramures, Salaj, Bistrita-Nasaud, Cluj, Bihor, Suceava); Center (Sibiu, Harghita, Mures, Alba, Brasov, Covasna); West (Munedoara, Arad); South Oltenia (Dolj, Gorj, Valcea), Muntenia region (Calarasi, Arges), South-East (Tulcea, Braila, Constanta, Galati). ^b^ Low-grade includes ASC-US and LSIL cytology. High-grade includes AGC, ASC-H, and HSIL cytology. Abbreviations: ASC-US – Atypical squamous cells of undetermined significance; ASC-H – Atypical squamous cells – cannot exclude HSIL; LSIL – Low grade squamous intraepithelial lesion; HSIL – High grade squamous intraepithelial lesion; AGC – Atypical Glandular Cells not otherwise specified; NILM – negative for intraepithelial lesion or malignancy; hrHPV – high-risk Human papillomavirus.

### Region

Nearly half of the participating women were from the North region of Romania (892; 43.2%), were we also found the highest age-standardized hrHPV prevalence (19.2%; 95% CI: 16.5–22.3%). The lowest age-adjusted hrHPV prevalence was found among women from the Center region (13.0%; 95% CI: 9.9–17.0%).

Compared to women living in the North region, women from the Center (OR = 0.65; 95% CI: 0.47–0.89) and South (OR = 0.62; 95% CI: 0.45–0.86) had significantly lower likelihood of hrHPV positivity. There was no significant difference between North and West regions. After adjusting for prior cervical cytology test, the associations of region with hrHPV positivity were no longer statistically significant (Table 3) compared to the North, due to the observation that only 6% of women in the North had prior cervical screening, while 61.7%, 88.7%, and 75.8% of women in the Center, West, and South had undergone previous cytological testing by the national cervical cancer program.

### Urban versus rural living area

A total of 1141 (55.4%) women were from urban (city) areas. The hrHPV prevalence for urban areas was 20.0%; (95% CI: 16.8–23.6); In rural areas the hrHPV prevalence was 14.8%; (95% CI: 12.4–17.7).

Logistic regression models did not show a statistically significant association between hrHPV-positivity and living area.

### hrHPV screening and cytology co-testing

Out of 2060 participants, 1615 (53.5%) did not have prior cervical cancer screening in the past three years. Among those who did have prior cervical cancer screening, 953 (82.2%) had a Romanian ethnicity. Among the 955 women who were co-tested for hrHPV and cytology, we found that 188 (19.7%) were positive for at least one of the two tests; 66 (7.9%) had the screening combination of being hrHPV positive and NILM; 89 (9.3%) had a combination of being hrHPV negative and cytology positive test and finally 30 women (3.1%) were positive for both hrHPV and cytology (Supplementary Table 2).

By age group, there was no difference between a positive versus NILM cytology result. The highest proportion of positive cytology result was found among women at the age of 35–44 years with 14.1% (p = 0.72) (Supplementary Table 3). The age-standardized prevalence of positive cytology among hrHPV positive women was 31.1%; (95% CI: 19.9–44.3%) among the screened women (data not shown). There were no differences in cytology abnormalities regarding ethnicity or living area.

Low-grade cytology (ASC-US or LSIL) and high-grade cytology (AGC, ASC-H, or HSIL) were highly associated with hrHPV positivity: OR = 3.17; 95% CI: 1.65–6.08 and OR = 5.18; 95% CI: 2.74–9.79), respectively. Those with prior cytology examinations were more likely to have a positive hrHPV test (OR = 2.54; 95% CI:1.89 – 3.41) than women who were not previously tested.

## Discussion

This study contributes basic information about the hrHPV prevalence in Romania among subpopulations before the onset of hrHPV vaccination and routine primary hrHPV screening, allowing for further surveillance of changes in hrHPV prevalence. The hrHPV prevalence of women in Romania has been studied in a real-world screening population among women with various ethnic backgrounds, including regions outside the Bucharest capital. Ethnic Romanians, Russians, and Hungarians had the highest hrHPV prevalence among all ethnic groups. hrHPV prevalence was also higher among women living in the North and West regions and among women from urban living areas.

This study found an overall prevalence rate of prevalence rate of 16.9% (95% CI: 14.7–19.5%). The worldwide prevalence of HPV among women without cervical abnormalities is estimated to be 11–12%, with higher rates in sub-Saharan Africa (24%), Eastern Europe (21%), and Latin America (16%) [14]. Few studies have reported the hrHPV prevalence in Romania and these have in addition been specifically focused on the Northwest region where the Romanian screening program was piloted [4,8]. The hrHPV prevalence rates we observed from different regions of Romania found in this study are comparable to other nearby European countries as: Poland 16.6%, Czech Republic 18.2%, Slovenia 12.9%, and Greece 12.7% [15,16,17,18]. Nevertheless, compared to other countries in Europe, Romania’s hrHPV prevalence is one of the highest after Czech Republic, Croatia, Estonia, Lithuania, Bulgaria, and Russia [15,19,20,21,22]. Other parts of Europe have a much lower hrHPV prevalence, in the order of 2–10% in Northern and Western European countries [14,23,24].

The higher hrHPV prevalence found among women belonging to the youngest age groups (less than 45 years old), is concerning. Cervical cancer is the most common cause of death by cancer in Romanian women aged 15–44 years [1,25]. This may be due, in part, to lack of a national cervical cancer screening program before 2012 and low participation rate for opportunistic screening [4]. In addition, known and widely recognized cervical cancer risk factors [26] may explain the high hrHPV prevalence in Romania. A high percentage of ethnic Romanian women have reported experiencing first intercourse at an early age, non-barrier intercourse, and more sexual partners than previous generations [24,25,27].

There are no available statistics on nationwide program attendance in Romania’s entirety since 2012. As a country with an over 10% ethnic minority population [6], this study addresses the knowledge gap regarding hrHPV prevalence among ethnic minorities in Romania. The highest hrHPV prevalence was found among Russian women and the lowest, amongst Roma and Ukrainian women. The hrHPV prevalence among ethnic Romanian women was 17.9%. The low hrHPV prevalence found among Roma women can partly be explained, we suggest, by the fact that many Roma in Romania live segregated from the main population, and often in small and closed communities [8,27]. Another study has shown that the vast majority of Roma women have one or two sexual partners during their lifetime [8,28]. Our study also found that when Roma women are screened for cervical cancer, they do so with a family doctor rather than a public or private hospital. The high hrHPV prevalance among Russian-Romanian women is in concordance with Russia’s comparatively high hrHPV prevalence among European countries [29].

Among Hungarians, we found a hrHPV prevalence of 16.6% which is similar to another Hungarian study (17.6%) [30]. This value could be explained by the fact that Hungarians are the largest minority being integrated in Romanian society, living mostly in urban areas [31]. Half of the Hungarians in our study also lived in cites, and their exposure to high hrHPV risk factors are likely similar to the Romanian population. Little information in the literature is available on hrHPV prevalence in Slovakia, however the hrHPV prevalence found in our study was 13.1%.

The late organizing of screening programs is common in many Central and Eastern European countries, and have common features such as a high cervical cancer incidence, mortality, and inequities in cervical cancer screening and sexual health education [32,33]. Inequalities in cervical cancer screening include differences in access to screening services and socioeconomic/demographic status [33]. Personal invitation to cervical screening plays a major role [33]. Our study found that a higher proportion of ethnic Romanians had prior cervical cancer screening experiences (86.5% compared to 78.4% in the study overall). The study also found that women in Romania from regions with high hrHPV prevalence (the Center, West, and South) had a lower proportion of prior cervical cancer testing than the North. Among women who did respond to the hrHPV screening service, a larger proportion of Romanians were tested at public hospitals (43.4% of ethnic Romanians) while 71.0% of Roma women tested at their family doctors. A cross-sectional study using a structured questionnaire among Roma and Romanian women found that for both ethnic groups, the main barrier for screening attendance were a lack of awareness about the screening program’s existence and also a lack of money to follow-up positive test results and not being included in the free-of-charge in Romania screening program [28].

Comparable to hrHPV positivity, the prevalence of positive cytology among women co-tested in our study was also 12.5%, which is higher than the positive cytology prevalence in Western Europe, ranging from 1.8% in Netherlands to 9.6% in France [16,18 34,35,36,37,38,39,40]. Both the higher prevalence of positive hrHPV and abnormal cytology tests are likely due to lack of regular cervical cancer screening, which is related to the relatively late introduction of cervical cancer screening programs in Romania [33].

This study has key strengths, including a diverse sample of women in Romania. To our knowledge, this is the first study to explore the hrHPV prevalence in Romania within varied ethnicities, regions, and living areas. While there have been no nationwide calculations of hrHPV prevalence in the general Romanian population, the hrHPV prevalence in Romania has been estimated to be between 10% to 48.4%, according to the proportion of women with normal to HSIL cytology [2,5]. In exploring subpopulation demographics, we were able to identify targetable factors that influence the burden of cervical cancer in a high cervical cancer incidence and mortality country. Limitations of this study include a lack of detailed data on other important hrHPV-related factors, such as age at onset of sexual activity, and number of sexual partners. However, the material presented in this study provides an outlook on hrHPV in a high cervical cancer rate country, providing a foundation for a more detailed investigation of the high burden of cervical cancer in Romania as well as a baseline hrHPV prevalence for future hrHPV vaccination programs. Finally, the sample size of Ukranian women was limited to only 24 women, reducing the power of correlational findings with HPV positivity for this ethnic group.

To enhance women in Romania’s screening uptake, efforts should be made to: 1) increase participation in the national screening program for women in the North of Romania, 2) women’s knowledge of the free-of-charge Romanian cervical cancer screening system for all ethnic groups, and 2) access to culturally representative trained health professionals, particularly for isolated communities and rural living areas with more limited access to screening. Similarly, health professionals in Romania need to take an active role in offering screening during health encounters, and providing education about sexual health communication with young women who are at highest risk for hrHPV infection.

While HPV prevalence is necessary to Identifying high-risk areas for cervical cancer in Romania, it is only the first step towards equity in cervical cancer screening. Women who received a positive HPV test and abnormal cytology in this study are undergoing clinical follow-up for screening and/or colposcopy and biopsy. Precancer and cancer outcome data are being collected and will be assessed in future studies. Stratification by HPV and cervical cytology screening result are necessary to assess cervical cancer risk, and will be done to determine the optimal approach to define Romanian cervical cancer screening guidelines.

The WHO recommends routine vaccination of girls 9–13 years old [41]. In an ethnically diverse country like Romania, where large proportions of the population live in closed communities or areas with limited access to health services, an outreach delivery strategy may be appropriate to ensure equitable vaccination opportunities for all adolescent girls. Education about HPV risk and the HPV vaccine will be the biggest barriers in introducing the HPV vaccination in Romania. In a cross-sectional survey study of 454 anonymous Romanian women, only 62.3% claimed to have heard about HPV vaccine, and 50.7% had a positive attitude towards it [42]. The barriers identified were fear of vaccine side effects, a perception of vaccination being risky, and costs of the vaccine. Moreover, there were deficiencies in knowledge for risks factors for HPV infection, such as early onset of sexual activity. While HPV vaccination does not replace routine cervical cancer screening, lack of awareness about HPV is a barrier for cervical cancer screening and prevention that can be improved via public health initiatives. In the context of vaccine delivery to remote areas, outreach strategy may require health care workers to leave their usual facility to transport and deliver immunization services in a variety of fixed or mobile sites in remote areas, a strategy recommended by the WHO [41].

## Conclusions

In conclusion, we observed a high prevalence of hrHPV, consistent with the high incidence of cervical cancer in the Romania. The hrHPV prevalence was highest in Russian, Romanian, and Hungarian ethnicity, for the North or West region, and for women living in urban living areas. This is the first study showing hrHPV prevalence among different ethnic groups living in Romania. Findings from this study are important in informing the national cervical screening program in Romania, providing specific hrHPV prevalence rates for actionable public health intervention.

## Disclaimer

Where authors are identified as personnel of the International Agency for Research on Cancer/World Health Organization, the authors alone are responsible for the views expressed in this article and they do not necessarily represent the decisions, policy or views of the International Agency for Research on Cancer/World Health Organization.

## Additional Files

The additional files for this article can be found as follows:

10.5334/aogh.2502.s1 Supplementary Table 1. Age groups by ethnicity among all Romanian women hrHPV screened for cervical cancer.

10.5334/aogh.2502.s2 Supplementary Table 2. Comparison between the cytological results and the hrHPV infection among Romanian women.

10.5334/aogh.2502.s3 Supplementary Table 3. Prevalence of hrHPV infection and prevalence of abnormal cytological results.

10.5334/aogh.2502.s4 Supplementary Figure 1. hrHPV screening location for each ethnic group in Romania.
