# Supplementary material for: HPV Testing for Cervical Cancer in Romania: High-Risk HPV Prevalence among Ethnic Subpopulations and Regions
Source: Ann Glob Health. 2019 Jun 20;85(1):89. doi: 10.5334/aogh.2502 (PMC6634611; doi:10.5334/aogh.2502)
Supplement: Supplementary Table 1. — Age groups by ethnicity among all Romanian women hrHPV screened for cervical cancer. [file agh-85-1-2502-s1.pdf]

Supplementary table 1. Age groups by ethnicity among all Romanian women hrHPV screened for cervical cancer

	Ethnicity						
	**Russian**	**Hungarian**	**Roma**	**Romanian**	**Slovakian**	**Ukrainian**	**p**
**Mean age**							
	42.7 +/-8.8	40.06 +/-9.8	40.13+/-9.0	42.82+/-10.0	43.8+/-9.1	41.0+/-9.3	0.02
**Age groups***							
≤ 34	22 (14.8%)	19 (30.7%)	24 (19.3%)	334 (21.4%)	13 (15.3%)	7 (29.2%)	0.01
35-44	76 (51.0%)	22 (35.5%)	29 (39.5%)	572 (35.5%)	36 (42.4%)	10 (41.7%)	
45-54	29 (19.5%)	18 (29.0%)	28 (32.3%)	450 (27.9%)	22 (25.9%)	5 (20.8%)	
55+	22 (14.8%)	3 (4.8%)	11 (8.9%)	245 (15.2%)	14 (16.5%)	2 (8.3%)	
Total	149 (100%)	62 (100%)	124 (100%)	1611(100%)	85 (100%)	24 (100%)	

* 5 persons with missing ages were excluded from this table.
Abbreviations: ASC-US - Atypical squamous cells of undetermined significance; ASC-H - Atypical squamous cells – cannot exclude HSIL; L-SIL - Low grade squamous intraepithelial lesion; H-SIL - High grade squamous intraepithelial lesion; AGC-NOS - Atypical Glandular Cells not otherwise specified; NILM - negative for intraepithelial lesion; hrHPV – high-risk Human Papillomaviirus;
