# Supplementary material for: HPV Testing for Cervical Cancer in Romania: High-Risk HPV Prevalence among Ethnic Subpopulations and Regions
Source: Ann Glob Health. 2019 Jun 20;85(1):89. doi: 10.5334/aogh.2502 (PMC6634611; doi:10.5334/aogh.2502)
Supplement: Supplementary Table 2. — Comparison between the cytological results and the hrHPV infection among Romanian women. [file agh-85-1-2502-s2.pdf]

Supplementary table 2. Comparison between the cytological results and the hrHPV infection among Romanian women

Cytological diagnosis*	n	hrHPV-positive		hrHPV-negative	
		n	%	n	%
NILM	833	66	7,9	767	92,1
ASC-US	58	11	19,0	47	81,0
ASC-H	31	8	25,8	23	74,2
AGC	21	4	19,0	17	81,0
LSIL	4	2	50,0	2	50,0
HSIL	5	5	100,0	0	0,0
Unsatisfactory	3	3	100,0	0	0,0
TOTAL	955	99	85,2	856	14,8

Abbreviations: ASC-US - Atypical squamous cells of undetermined significance; ASC-H - Atypical squamous cells – cannot exclude HSIL; L-SIL - Low grade squamous intraepithelial lesion; H-SIL - High grade squamous intraepithelial lesion; AGC-NOS - Atypical Glandular Cells not otherwise specified; NILM - negative for intraepithelial lesion or malignancy; hrHPV – high-risk Human Papillomavirus;
