# Supplementary material for: HPV Testing for Cervical Cancer in Romania: High-Risk HPV Prevalence among Ethnic Subpopulations and Regions
Source: Ann Glob Health. 2019 Jun 20;85(1):89. doi: 10.5334/aogh.2502 (PMC6634611; doi:10.5334/aogh.2502)
Supplement: Supplementary Table 3. — Prevalence of hrHPV infection and prevalence of abnormal cytological results. [file agh-85-1-2502-s3.pdf]

Supplementary table 3. Prevalence of hrHPV infection and prevalence of abnormal cytological results

| | HPV test (n= 2,060) | | | Cytology test (n= 952)[a] | | |
|---|---|---|---|---|---|---|
| | | | | Positive | | |
| | Positive | Negative | p | ASCUS, ASCH, AGC-NOS, L-SIL or H-SIL | NILM | p |
| | n (%) | n (%) | | n (%) | n (%) | |
| **Age groups (years)[b]** | | | | | | |
| <=34 | 99 (23.1) | 330 (76.9) | <0.001 | 18 (11.4) | 140 (88.6) | 0.72 |
| 35-44 | 102 (13.3) | 663 (86.7) | | 50 (14.1) | 304 (85.9) | |
| 45-54 | 64 (11.3) | 500 (88.7) | | 36 (12.1) | 262 (87.9) | |
| >=55 | 38 (12.8) | 259 (87.2) | | 15 (10.6) | 127 (89.4) | |
| **Ethnicity** | | | | | | |
| Other ethnicity[c] | 54 (12.1) | 391 (87.9) | <0.001 | 27 (9.3) | 263 (90.7) | 0.06 |
| Romanian | 251 (15.5) | 1364 (84.5) | | 92 (13.9) | 570 (86.1) | |
| **Regions** | | | | | | |
| North | 161 (18.1) | 731 (82.0) | 0.003 | 19 (8.1) | 213 (91.8) | 0.001 |
| Center | 58 (12.3) | 415 (87.7) | | 30 (9.6) | 282 (90.4) | |
| West | 26 (14.0) | 160 (86.0) | | | | |
| South | 60 (11.8) | 449 (88.2) | | 70 (17.2) | 338 (82.8) | |
| **Environment** | | | | | | |
| Rural | 174 (15.3) | 967 (84.8) | 0.49 | 64 (12.9) | 429 (86.5) | 0.22 |
| Urban | 130 (14.2) | 788 (85.8) | | 55 (12.0) | 404 (88.0) | |
| **Total** | 304 (14.8) | 1756 (85.2) | | 119 (12.8) | 833 (87.2) | |

[a]Excludes those with previously taken cytology tests and three unsatisfactory cytology results

[b]Excludes 5 missing ages

[c]Other ethnicity = Russian, Roma, Hungarian, Slovakian, and Ukrainian

Abbreviations: ASC-US - Atypical squamous cells of undetermined significance; ASC-H - Atypical squamous cells – cannot exclude HSIL; L-SIL - Low grade squamous intraepithelial lesion; H-SIL - High grade squamous intraepithelial lesion; AGC-NOS - Atypical Glandular Cells not otherwise specified; NILM - negative for intraepithelial lesion or malignancy; hrHPV – high-risk Human Papillomavirus.
