# Supplementary figures and images for: HPV Testing for Cervical Cancer in Romania: High-Risk HPV Prevalence among Ethnic Subpopulations and Regions
Source: Ann Glob Health. 2019 Jun 20;85(1):89. doi: 10.5334/aogh.2502 (PMC6634611; doi:10.5334/aogh.2502)

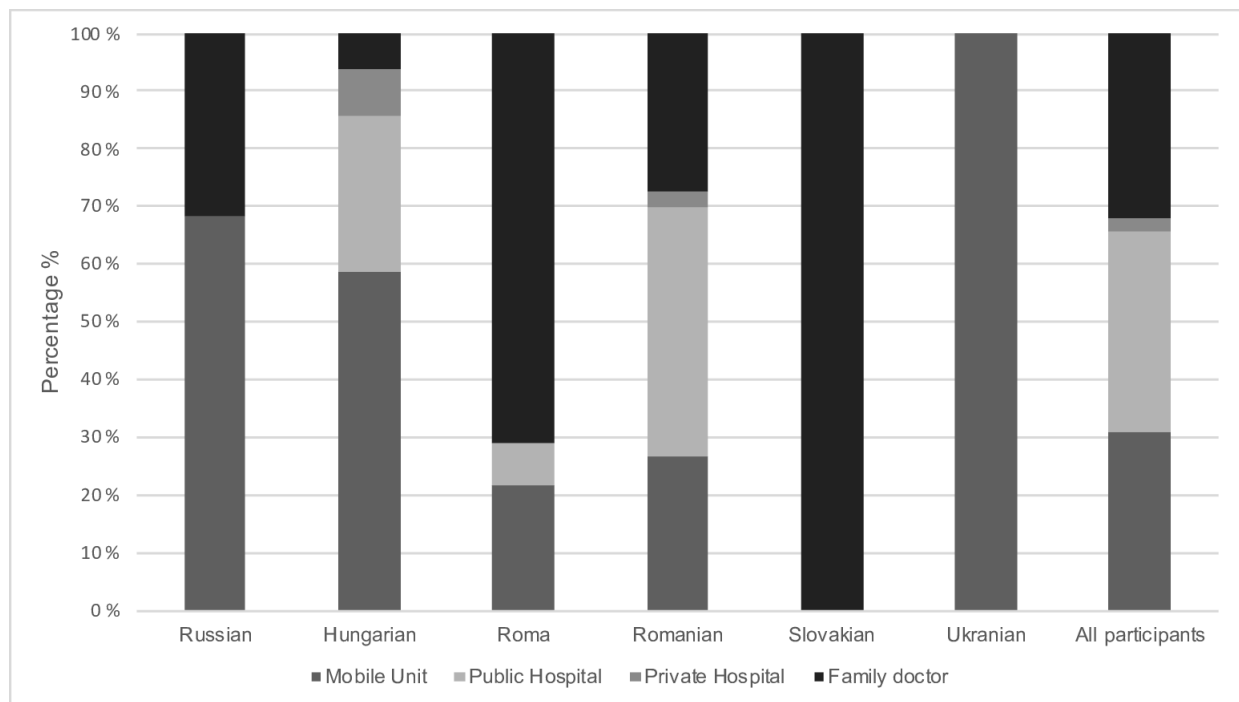

Supplementary Figure 1. hrHPV screening location for each ethnic group in Romania.

Supplement: Supplementary Figure 1. — hrHPV screening location for each ethnic group in Romania. [file agh-85-1-2502-s4.pdf]
